# Adolescents’ experiences of fluctuating pain in musculoskeletal disorders: a qualitative systematic review and thematic synthesis

**DOI:** 10.1186/s12891-020-03627-1

**Published:** 2020-10-02

**Authors:** Sonia Khanom, Janet E. McDonagh, Michelle Briggs, Ebru Bakir, John McBeth

**Affiliations:** 1 Centre for Epidemiology Versus Arthritis , School of Biological Sciences, Faculty of Biology, Medicine and Health, University of Manchester, 2.706 Stopford Building, Oxford Road, Manchester, M13 9PT UK; 2 NIHR Manchester Biomedical Research Centre, Manchester University NHS Foundation Trust, Manchester, UK; 3 Division of Nursing, Midwifery and Social Work, School of Health Sciences, Faculty of Biology, Medicine and Health, University of Manchester, Manchester, UK

**Keywords:** Adolescence, Juvenile arthritis, Chronic pain, Patient experience, Qualitative research

## Abstract

**Background:**

Adolescents with chronic musculoskeletal pain experience daily fluctuations in pain. Although not all fluctuations are bothersome, pain flares are a distinct type of symptom fluctuation with greater impact. Since literature on the experience of pain flares is non-existent, the aim of this review was to (i) synthesise the qualitative literature on adolescents’ experiences of fluctuating pain in musculoskeletal disorders in order to (ii) identify knowledge gaps to inform future research on pain flares.

**Methods:**

Electronic databases (CINAHL, MEDLINE, EMBASE, PsycINFO), grey literature and reference lists were searched from inception to June 2018 for qualitative studies reporting adolescents’ experiences of pain. Comprehensiveness of reporting was assessed using the Consolidated Criteria for Reporting Qualitative Health Research. Studies were analysed using thematic synthesis.

**Results:**

Of the 3787 records identified, 32 studies (*n* = 536) were included. Principal findings were synthesised under three key themes: 1) symptom experience, 2) disruption and loss, and 3) regaining control. The first theme (symptom experience) describes adolescent’s perception and interpretation of pain fluctuations. The second theme (disruption and loss) describes the physical, social and emotional constraints faced as a result of changes in pain. The third theme (regaining control) describes coping strategies used to resist and accommodate unpredictable phases of pain. Each theme was experienced differently depending on adolescents’ characteristics such as their developmental status, pain condition, and the duration of the pain experience.

**Conclusions:**

Adolescents with chronic musculoskeletal pain live with a daily background level of symptoms which frequently fluctuate and are associated with functional and emotional difficulties. It was not clear whether these symptoms and challenges were experienced as part of ‘typical’ fluctuations in pain, or whether they reflect symptom exacerbations classified as ‘flares’. Further research is needed to explore the frequency and characteristics of pain flares, and how they differ from their typical fluctuations in pain. The review also highlights areas relating to the pain experience, symptom management and health service provision that require further exploration to support more personalised, tailored care for adolescents with chronic musculoskeletal pain.

## Key messages

- Chronic musculoskeletal pain frequently fluctuates within and across days
- Pain flares are distinct from typical, everyday fluctuations in pain
- Research is required to explore the characteristics of pain flares that distinguish it from other fluctuations

## Background

Musculoskeletal pain is responsible for approximately 11% of primary care visits of young people aged 10–24 years [1, 2], and is the most common reason for which they are referred to paediatric rheumatology services [3]. While some pain may have identifiable pathology, the majority of young people do not have inflammatory or other obvious disease processes to explain their pain [4]. In juvenile idiopathic arthritis (JIA), even when there is effective control of disease activity and inflammation young people continue to report significant pain [5–7]. Similarly, in chronic idiopathic pain syndromes (CIPS), which include complex regional pain syndrome and juvenile fibromyalgia [8], there is a lack of consistent correlation between pain and tissue pathology.

Musculoskeletal pain, both inflammatory and non-inflammatory, fluctuates within and across days [9–13]. Not all fluctuations are considered important by individuals, however ‘pain flares’ are a distinct type of symptom fluctuation with greater impact [14–18]. There is a lack of research into the definition and experiences of pain flares in young people, but studies report periods of significantly increased pain which affect quality of life [19–21]. For example, diary studies report that 66 and 67% of young people with in JIA and juvenile fibromyalgia, respectively, experienced daily changes in pain intensity ≥10 units on a 0–100 visual analogue scale [12, 13], with greater changes in pain being associated with lower quality of life [12, 13, 22]. This may present challenges for adolescents at this stage of their development which includes brain maturation, emergence of abstract thinking, establishing relationships outside the family and the gradual process of achieving independence from parents [17].

While findings show that periods of increased pain are commonly experienced among adolescents, little remains known about the nature and experience of pain flares and how they differ from other fluctuations in pain. Due to the paucity of information on living with pain flares, this review aims to identify and synthesise existing qualitative literature on fluctuating musculoskeletal pain as a starting point, in order to identify knowledge gaps to inform future research of pain flares. Noblit and Hare [23] describe two types of qualitative syntheses: integrated reviews that aim to summarise or ‘aggregate’ findings, and interpretative reviews which aim to interpret findings and generate theory [24, 25]. This review is an aggregated review which seeks to address the following question: what are adolescents’ experiences of pain fluctuations in daily life?

## Methods

This systematic review followed the Enhancing Transparency in Reporting the Synthesis of Qualitative Research framework [26]. No review protocol has been previously published.

### Eligibility criteria

To be included, the studies 1) used a qualitative design, 2) included participants with JIA or CIPS (CIPS incudes fibromyalgia, complex regional pain syndrome or chronic pain (pain ≥3 months) that does not have a definite origin like cancer), 3) included participants aged 10–19 years, or with a mean age within the 10 to 19 year range, as based on the World Health Organisation definition of adolescence [27], or with a mean age within the 10 to 19 year range, 4) reported participants’ own experiences of pain, and 5) were reported in English.

### Data sources and searches

Four electronic databases (CINAHL, MEDLINE, EMBASE, PsycINFO) were searched from inception to June 2018, and supplemented with searches of grey literature (OpenGrey, Scopus) and reference lists of relevant studies and reviews. The Population, Exposure and Outcome (PEO) framework [28] was used to direct the development of the search terms: ‘Population’ was adolescents with JIA or CIPS, ‘Exposure’ was pain, and ‘Outcome’ was experience (Table 1). See Additional file 1 for the full search strategy and hits.

**Table 1:** Database search terms using the Population, Exposure and Outcome (PEO) framework

PEO tool	Search terms
Population	exp arthritis, juvenile OR juvenile chronic arthritis OR juvenile idiopathic arthritis OR juvenile rheumatoid arthritis OR musculoskeletal pain OR chronic idiopathic pain OR idiopathic pain OR juvenile fibromyalgia or fibromyalgia OR diffuse* pain OR diffuse* idiopathic pain OR widespread pain OR generalised pain OR pain amplification OR complex regional pain syndrome or CRPS OR local* pain OR local* idiopathic pain AND adolescen* OR child* OR young person OR young people OR youth OR p*ediatric
Exposure	Pain
Outcome	experience* OR view OR percep* OR perspective OR concern* OR attitude* OR feel* OR perceive OR belie* OR opinion

### Study selection

Titles and abstracts from the searches were screened by SK against the inclusion criteria. The selected full-text articles were independently reviewed by SK and EB, of which ineligible articles were excluded. Disagreements were resolved by discussion between SK and EB.

### Quality appraisal

The transparency of reporting was assessed using the Consolidated Criteria for Reporting Qualitative Health Research (COREQ) framework [29]. All disagreements were resolved via discussion.

### Data extraction

Data were extracted on the year of publication, country, participant characteristics, data collection, analysis methods, study purpose, and whether they included data on fluctuating pain.

### Data synthesis

The data were analysed using thematic synthesis outlined by Thomas and Harden [30]. Accordingly, all text under the ‘results/findings’ or ‘discussion/conclusion’ section of each article were extracted. Starting with one article, SK performed line-by-line coding of the findings. At least one code was given to all statements relating to pain; example codes included ‘cause of pain unknown’ ‘no sign of swelling’ and ‘doctors do not understand’. After coding the first paper, a ‘bank’ of codes was generated which identified aspects of the pain experience and discussed with all authors. Subsequent articles were analysed similarly with new codes being added to the existing bank of codes. Codes were then organised into descriptive themes by grouping the codes based on their similarities and differences. This process involved repeated reference back to the original papers to ensure interpretations were grounded in the words of participants. Examples themes included ‘behaviour of health professionals’ and ‘feeling disbelieved’. Descriptive themes were then further interpreted to develop analytical themes through discussion with all authors. This involved a process of examining and discussing patterns and relationships across descriptive themes, which led to the emergence of more abstract messages and themes that go beyond the content in the original studies. The final themes are discussed below.

## Results

### Search results

**Fig. 1:**
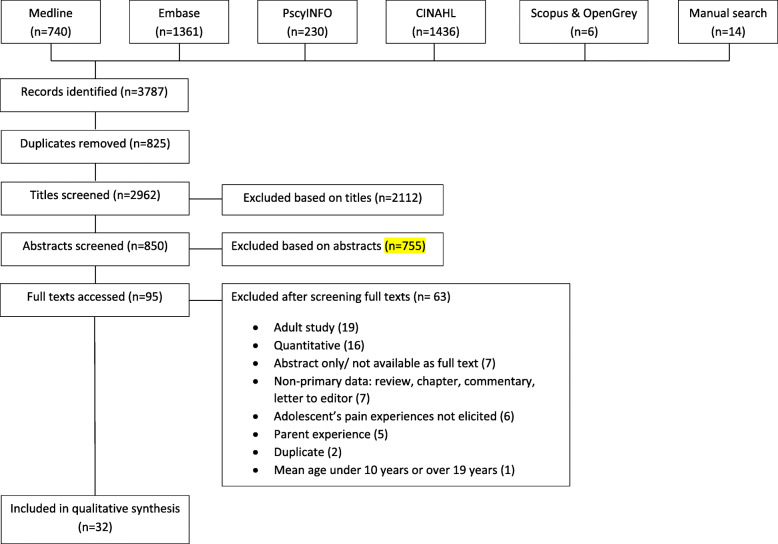
Study identification and selection process

A total of 3787 studies were identified (Fig. 1). These were imported into Endnote X8 and the full texts of 95 papers were retrieved and screened for inclusion. Thirty-two studies fulfilled the inclusion criteria and were included in the review (Table 2).

**Table 2:** The characteristics of the included studies in the review, presented in order of year they were published

Author (year)	Country	Participants	Age (years)	Gender	Data collection	Data analysis	Principal research topic	Discussed pain fluctuations	Problems with extracting data
1. Beales et al. (1983) [31]	UK	JIA *n* = 75	7–17	48 female 27 male	Face to face interview	–	Children and adolescents with JIA beliefs about the physical nature of their illness and treatment	Yes	No
2. Beales et al. (1983) [32]	UK	JIA *n* = 39	6–17	- female - male	Face to face interview	–	The meaning children and adolescents with JIA attribute to joint sensations and pain	No	No
3. Berry et al. (1993) [33]	US	JIA *n* = 54	6–17 (M = 12 years)	40 female 14 male	Face to face interview	Content analysis	Children and adolescents with JIA understanding and conceptualisation of illness.	No	No
4. Woodgate (1998) [34]	Canada	JIA *n* = 4 Other: diabetes *n* = 11, asthma *n* = 5, Crohn’s disease *n* = 2, ulcerative colitis *n* = 1 Total *n* = 23	13–16*	12 female* 11 male*	Face to face interview	Grounded theory analysis	Adolescents’ descriptions of chronic illness experiences	Yes	No
5. Barlow el al. (1999) [35]	UK	JIA *n* = 10 Other: health professionals *n* = 7, parents *n* = 13 Total *n* = 30	8–15	5 female 5 male	Focus group	Thematic analysis	The needs and preferences of children and adolescents with JIA, and their perspectives on psychoeducational interventions	No	No
6. Sällfors et al. (2001) [36]	Sweden	JIA *n* = 22	6–17 (median = 15 years)	16 Female 6 male	Face to face interview	Grounded theory	How children and adolescents with JIA cope with chronic pain in daily life	Yes	No
7. Britton & Moore (2002) [37]	UK	JIA *n* = 9 Other: parents *n* = −, siblings *n* = − Total *n* = −	7–13 years	9 Female 0 male	Questionnaire, face to face interviews, written diary, video diary	Grounded theory analysis	The experiences of families of young people with JIA	Yes	No
8. Britton & Moore (2002) [38]	UK	JIA *n* = 9 Other: parents *n* = −, siblings *n* = − Total *n* = −	7–13 years	9 Female 0 male	Questionnaire, face to face interviews, written diary, video diary	Grounded theory analysis	The families’ perspectives about daily exercises, splinting and medication in JIA	Yes	No
9. Sällfors et al. (2002) [39]	Sweden	JIA *n* = 22	6–17 (male median age = 11 years, female median age = 16 years)	16 Female 6 male	Face to face interview	Grounded theory analysis	Life situation and psychosocial processes of living with chronic pain in JIA	Yes	No
10. Kyngäs (2004) [40]	Finland	JIA *n* = 6 Other: diabetes *n* = 14, asthma *n* = 12, epilepsy *n* = 8 Total *n* = 40	13–17*	26 Female* 14 male*	Face to face interview	Content analysis	Support network of adolescents with a chronic disease	Yes	Yes- unclear which themes derived from young people with JIA
11. Batthish et al. (2005) [41]	Canada	JIA *n* = 14 Other: parents *n* = 11, Total *n* = 25	6–18 (*M* = 12 years)	8 Female 6 male	Face to face interview	Content analysis	Perceptions of active disease among children and adolescents with SO-JIA and their parents	No	No
12. Pelaez-Ballestas et al. (2016) [42]	Mexico	JIA n = 6 Other: parents *n* = 16, Total *n* = 22	*M* = 13 years	4 Female 2 male	Face to face interview	Interpretative grounded theory methodology and explanatory models	Experiencing JIA within a specific cultural context	No	No
13. (Guell, 2007) [43]	Germany and UK	JIA *n* = 4 Other: parents *n* = −, siblings *n* = −, health professionals *n* = − Total *n* = −	7–16	3 Female 1 male	Face to face interview, observation	Ethnographic approach	The everyday life and coping of children and adolescents with JIA	Yes	No
14. Stinson et al. (2007) [44]	UK	JIA *n* = 36	12–20 (*M* = 15 years)	24 Female 12 male	Face to face interview and focus group	Thematic analysis	Self-management needs of adolescents with JIA	No	No
15. Fuchs et al. (2008) [45]	The Netherlands	JIA *n* = 1	18	1 Female	Face to face interview	The self-confrontation method	The personal experience and feelings of an adolescent with JIA	No	No
16. De Monte et al. (2009) [46]	Australia	JIA *n* = 13	8–16 (*M* = 11 years)	11 Female 2 male	Face to face interview	Thematic analysis	Children and adolescents with JIA perceptions about their participation in home exercise programmes	No	No
17. Meldrum et al. (2009) [47]	US	CRPS *n* = 5 Fibromyalgia *n* = 5 Other: headaches *n* = 31, functional neurovisceral pain disorder *n* = 20, myofascial pain *n* = 18 (25 young people reported more than one type of pain). Total *n* = 53	10–17*	36 Female* 17 male*	Face to face interview	Grounded theory and narrative analysis	The experiences and impact of chronic or recurrent pain on children and adolescents	Yes	Yes- unclear which themes derived from young people with CRPS/ fibromyalgia. However, authors reported no significant differences between responses based on the type of pain experienced
18. Sällfors (2009) [48]	Sweden	JIA *n* = 6	14–17	6 Female 0 male	Face to face interview written diary	Grounded theory	Female adolescents’ daily living with chronic arthritis	Yes	No
19. Secor-Turner et al. (2011) [49]	US	JIA *n* = 5 Other: young adults with JIA *n* = 5 Total *n* = 10	14–21 (*M* = 16 years)	3 Female 2 male	Face to face interview and focus group	Content analysis	Challenges that adolescents experience while living with JIA from the perspective of youth and young adults with JIA.	Yes	No
20. Gorodzinsky et al. (2013) [50]	US	Chronic musculoskeletal pain *n* = 2 Other: functional abdominal pain *n* = 4, gastritis *n* = 1, post-concussive syndrome *n* = 1, chronic migraines *n* = 1, Siblings *n* = 9 Total *n* = 17	12–18 years (*M* = 15 years)	7 Female 1 male	Face to face or telephone interview	Delphi coding procedure	Experiences of children and adolescents with chronic pain and their siblings, and how pain influences family dynamics	No	Yes- unclear which themes derived from young people with chronic musculoskeletal pain
21. Gray et al. (2013) [51]	UK	JIA *n* = 19 Other: Other arthritis *n* = 2 Total *n* = 21	11–19*	17 Female* 4 male*	Blogs	Thematic analysis and corpus linguistics	The relationship between identity and medication use amongst adolescents with arthritis, and the role of pharmacy in delivering services to this group.	Yes	No
22. Jacobson et al. (2013) [52]	US	JIA *n* = 18, Fibromyalgia *n* = 1, CRPS *n* = 1 Other: migraines *n* = 4, chronic headaches *n* = 3, abdominal pain *n* = 2, chronic foot pain *n* = 1, progressive pseudorheumatoid chondrodysplasia *n* = 1, chronic lower back pain *n* = 1 Total *n* = 34	8–18 (*M* = 14 years)*	28 Female* 6 male*	Face to face interview	Thematic and content analyses	The performance and content validity of PROMIS paediatric measures among children and adolescents with chronic pain conditions	Yes	Yes- unclear which themes derived from young people with JIA/ CRPS/ Fibromyalgia. However, authors reported no evidence of differences across diagnostic groups.
23. Tong et al. (2013) [53]	Australia	JIA *n* = 13 Other: parents *n* = 37 Total *n* = 50	14–19	9 Female 4 male	Face to face or telephone interview	Thematic analysis	Parental and adolescent perspectives on paediatric rheumatology care and service delivery	Yes	No
24. Guzman et al. (2014) [54]	Canada	JIA *n* = 9 Other: parents *n* = 23, health professionals *n* = 17 Total *n* = 49	16–23	7 Female 2 male	Face to face interview and focus group	Content analysis	Identifying clinical features most important for adolescents, parents and clinicians in the course of JIA	No	No
25. Cartwright et al. (2015) [55]	UK	JIA n = 10	13–17	7 Female 3 male	Face to face interview	Interpretative phenomenological analysis	Adolescents’ experiences of living with JIA and the process of adjustment	No	No
26. Condon et al. (2015) [56]	Ireland	JIA *n* = 26 Other: parents *n* = − Total *n* = −	3–18 (*M* = 11 years)	19 Female 7 male	Face to face interview	Qualitative descriptive approach	self-management needs and coping activities of children and adolescents with JIA and their parents	Yes	No
27. Jacobson et al. (2016) [57]	US	JIA *n* = 11, Fibromyalgia *n* = 4 Other: migraine *n* = 5, sickle cell disease *n* = 8, parents *n* = 14 Total *n* = 42	8–17	18 Female 10 male	Face to face interview, focus group	Content analysis	The conceptual scope and content validity of the PROMIS pain domain framework among children with chronic pain conditions	No	No
28. Race et al. (2016) [58]	Canada	JIA *n* = 23 Other: parents *n* = 29 Total *n* = 37	8–16 (*M* = 12 years)	15 Female 8 male	Face to face interview	Framework Analysis	Perspectives of children and adolescents with JIA and their parents about the barriers and facilitators to participation in physical activity	Yes	No
29. Suder (2016) [59]	US	CRPS *n* = 2 Amplified pain syndrome *n* = 1 Other: headaches *n* = 6, migraines *n* = 5, lower back pain *n* = 2, abdominal pain *n* = 2, wrist pain *n* = 1, ankle pain *n* = 1, chronic leg pain *n* = 1 (8 young people reported more than one type of pain). Total *n* = 10	14–17	3 Female 0 male	Face to face interview, visual depictions, researcher journaling	Thematic analysis	The lived experience of adolescents who live with chronic pain.	Yes	No
30. Sørensen et al. (2017) [60]	Norway	CRPS *n* = 4, Extreme muscle pain *n* = 2 Total *n* = 6	12–19	4 Female 2 male	Face to face interview	Hermeneutic analysis	Adolescents’ experiences of complex persistent pain and its impact on everyday life	Yes	No
31. Ghio et al. (2018) [61]	UK	JIA *n* = 20	11–16	13 Female 7 male	Face to face interview	Framework and content analysis	The suitability and validity of an illness questionnaire for use with adolescents with JIA	Yes	No
32. Modica et al. (2018) [62]	US	JIA *n* = 25 Other: parents n = 7 Total *n* = 32	13–20	- Female - male	Social media post	Sociolinguistics and semiotics	Experience of adolescents with systemic JIA and their parents based on their social media posts	No	No

*JIA* juvenile idiopathic arthritis

*CRPS* complex regional pain syndrome

- = unknown

* = did not specify age and sex of participants with JIA or CIPS

### Study characteristics

The 32 studies were published from 1983 to 2018 and conducted in 11 countries: United Kingdom [10], United States [8], Canada [4], Sweden [3], Australia [2], Finland [1], Germany [1], Ireland [1], Mexico [1], Norway [1], and The Netherlands [1]. Interviews were conducted in 29 studies, with 9 studies also combining interviews with focus groups, observations, questionnaires, researcher journaling, visual depictions and diaries. One study collected data using only social media posts, one using blog posts and one using focus groups. Eleven studies used a longitudinal approach where interviews/observations were collected at more than one time point.

In total 536 young people with JIA or CIPS participated in the studies, of which 509 had a diagnosis of JIA, and 27 with CIPS. Although all included studies reported adolescents’ experience of pain, only 19 studies provide insight into the impact of fluctuating pain on an individual’s life and lived experience. 21 studies included data from parents, siblings, health professionals and/or individuals with other chronic illnesses, but efforts were made to only extract data referring to or expressed by adolescents with JIA or CIPS. Ages of young people ranged from 3 to 23 years, but all studies averaged within the adolescent range.

### Comprehensiveness of reporting

The comprehensiveness of reporting was variable, with studies reporting between 10 and 28 of the 32 COREQ-checklist items (Table 3). Twenty-five studies provided details on at least 50% of the criteria.

**Table 3:** Transparency of reporting assessed studies using the COREQ framework

	References																													
Items	44	45	43	48	56	52	37	36	50	62	32	82	55	61	63	41	51	46	39	53	47	38	31	59	40	60	54	35	34	33
**Domain 1: Research team and reflexivity**																														
• Interviewer/facilitator	+	+	+	+	+	+	+	+	+	+	–	+	+	+	–	+	–	+	–	–	–	+	–	+	+	–	+	+	+	–
• Credentials	–	–	–	+	–	+	+	+	–	+	–	–	+	+	–	–	+	+	+	–	+	+	–	–	–	+	–	+	–	–
• Occupation	+	+	+	+	+	+	+	+	+	–	–	–	+	+	+	+	–	–	+	+	+	+	–	+	+	+	–	–	+	–
• Gender	–	–	+	+	–	+	+	+	+	+	–	–	+	+	+	+	+	+	+	+	+	+	+	+	+	+	+	+	+	+
• Experience and training	+	–	–	–	–	–	–	–	–	–	+	–	–	+	–	–	+	–	–	+	+	+	–	+	–	+	–	–	–	+
• Relationship established	+	–	–	+	–	–	–	–	–	–	+	–	+	–	–	+	–	–	–	–	–	–	–	–	–	–	+	+	+	+
• Participant knowledge of the interviewer	+	–	–	–	–	–	+	–	–	–	–	–	–	–	–	–	–	–	–	–	–	–	–	–	–	–	–	–	–	–
• Interviewer characteristics	–	–	–	–	+	–	+	+	–	–	–	–	+	–	–	–	–	–	–	–	–	+	–	–	+	–	–	–	+	–
**Domain 2: Study design**																														
• Methodological orientation and theory	–	–	+	+	+	+	+	+	+	–	+	+	+	–	+	–	+	+	+	–	+	+	–	+	–	+	–	–	+	–
• Sampling	+	+	+	+	+	+	+	+	+	+	+	–	+	+	+	+	+	+	+	+	+	+	+	+	+	+	+	+	+	+
• Method of approach	–	–	+	+	–	–	+	+	–	+	–	+	+	–	+	+	+	+	+	+	+	+	+	+	+	+	+	+	+	+
• Sample size	+	+	+	+	+	+	+	+	+	+	+	+	+	+	+	+	+	+	+	+	+	+	+	+	+	+	+	+	+	+
• Non-participation	–	–	+	–	+	–	–	–	–	+	–	–	+	–	–	+	+	–	–	+	+	–	–	+	–	–	+	+	+	+
• Setting of data collection	+	+	+	+	+	+	–	+	+	+	+	+	+	+	+	+	+	–	–	+	+	+	–	+	+	+	+	+	+	+
• Presence of nonparticipants	–	–	–	–	–	–	–	–	+	–	–	–	–	–	–	+	–	–	–	–	–	+	+	–	–	–	–	+	–	–
• Description of sample	+	+	+	+	+	+	+	+	+	+	+	+	–	+	+	+	+	+	+	+	+	+	+	+	+	+	+	+	+	–
• Interview guide	+	+	+	+	+	+	–	–	+	+	+	–	–	+	+	+	–	+	+	+	–	+	+	+	+	+	–	+	+	+
• Repeat interviews	–	–	–	+	–	–	+	+	–	–	–	+	+	–	+	+	+	+	–	–	–	+	–	–	–	–	–	+	–	–
• Audio/visual recording	–	–	+	+	+	+	+	+	+	+	+	+	+	+	–	+	+	+	+	+	+	+	+	+	+	+	+	+	+	+
• Field notes	–	–	–	+	+	+	–	–	–	–	–	+	+	+	–	+	–	–	+	–	+	+	+	+	+	–	–	+	+	+
• Duration	–	–	–	+	+	+	–	–	+	+	+	+	+	+	–	–	+	+	+	+	+	+	–	+	–	+	+	+	+	–
• Data saturation	–	–	–	+	+	+	–	–	+	–	–	–	–	–	–	–	–	+	–	–	+	+	+	–	+	–	+	+	–	–
• Transcripts returned	–	–	–	–	–	–	–	–	–	–		–	–	–	–	–	–	–	–	–	–	–	–	–	+	–	–	–	–	–
**Domain 3: analysis and findings**																														
• Number of data coders	–	–	+	–	+	–	+	+	+	–	–	+	–	+	–	+	–	–	+	+	+	+	+	+	+	–	+	+	+	+
• Description of the coding tree	+	+	+	–	+	+	–	–	+	–	+	–	–	+	+	+	–	+	+	+	+	+	+	+	+	+	–	+	–	+
• Derivation of themes	–	–	–	+	+	+	+	+	+	+	+	+	–	+	–	+	+	+	+	+	+	+	+	+	+	+	+	+	+	+
• Software	–	–	–	–	–	–	+	+	–	–	–	+	–	+	–	–	+	–	–	–	+	+	–	–	–	–	+	+	–	+
• Participant checking	–	–	–	–	+	–	–	–	–	–	–	–	–	–	–	+	–	–	–	–	–	+	–	–	–	–	–	–	–	+
• Quotations presented	–	–	+	+	+	+	+	+	+	+	+	+	+	+	+	+	+	+	+	+	+	+	–	+	+	+	+	–	+	+
• Data and findings consistent	+	+	+	+	+	+	+	+	+	+	+	+	+	+	+	+	+	+	+	+	+	+	+	+	+	+	+	+	+	+
• Clarity of major themes	+	+	+	+	+	+	+	+	+	+	–	+	+	+	+	+	+	+	+	+	+	+	+	+	+	+	+	+	+	+
• Clarity of minor themes	–	–	–	+	–	+	+	+	+	–	–	–	+	+	–	+	–	+	+	–	–	+	–	+	–	–	+	+	+	–

### Synthesis of studies

**Fig. 2:**
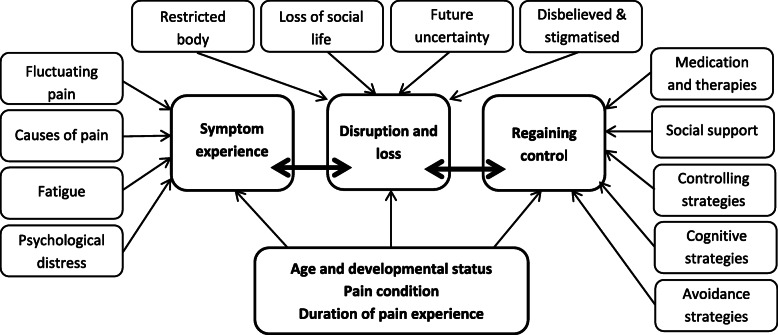
Themes illustrating adolescents’ experiences of fluctuating pain

Three themes emerged from the synthesis: symptom experience; disruption and loss; and regaining control. These themes can be seen to describe a journey through which the adolescent experiences fluctuating pain and associated symptoms, encounters the challenges to lifestyle that fluctuating pain presents, followed by employing coping strategies to regain a sense of control. This journey of experiencing pain is not a linear process and the themes are not mutually exclusive. Each stage is experienced differently depending on individual factors such as developmental status, pain condition, and duration of the pain experience (Fig. 2). Quotations corresponding to the themes are shown in Table 4.

**Table 4:** Quotations from the studies to support the themes

Analytical theme/subtheme	Quotations
**1. Symptom experience**	
• Pain	“It was a solid 7, like 24/7, so sometimes it would get up to a 10, like maybe like every other 2 days but that would still happen” (female, 17 years, CRPS) [59]
• Causes of pain	“I understand what it is and that it makes everything swell up and like hurt but some stuff I don’t know like why it suddenly can just start hurting for no reason*”* (female, 13 years, JIA) [61]
• Fatigue	“I think when I’m tired I can feel it (lower back pain) more and I get crankier and I feel like my back hurts “just leave me alone.” When my back injury was really bad I’d only get like a couple hours of sleep and then I’d be tired throughout the day” (female, 15 years, CIPS) [52]
• Psychological distress	“You get so disappointed, because suddenly you’re better... and then you’re worse again. All the time you’re hoping that you’ll be better… but sometimes you got worse” (participant with JIA) [39]
**2. Disruption and loss**	
• Restricted body	“It’s annoying like just not being able to do things and then having to think ahead about whether I do one thing then I can’t do another thing tomorrow. Not being able to participate in things at school and having to answer people’s questions is also a downside” (female, 16 years, JIA) [46]
• Loss of social life	“I feel I get left out from my friends and everything because I can’t do as many things as they do so they don’t even ask me if I want to come along” (female, 14 years, JIA) [34]
• Future uncertainty	“Like me in tall grass, like enemies around me, and… I don’t know where they are, when they’re going to attack” (participant with CIPS) [47]
• Disbelieved and stigmatised	“My peers thought it was strange that one day you arrive on crutches and the next you can hardly walk… and then you can walk normally… it didn’t make sense to them” (participant with JIA) [39]
**3. Regaining control**	
• Medication and therapies	“I had, like, a lot of pain in the morning, and then after, like, a lot of walking in the morning it sort of went away” (female, 14 years, JIA) [58]
• Social support	“They (parents) help me to take good care of my arthritis... sometimes I have very bad pains and my parents help me to stand them” (participant with JIA) [40]
• Adapting behaviours	“I have to watch what I eat or watch what activities I’m doing, planning ahead what I’m going to do for that week so I don’t – see, that’s the thing… I plan ahead more than I really want to” (participant with CIPS) [47]
• Cognitive strategies	“I prepare myself almost every day to wake up having excruciating pain” (female, JIA) [48]
• Avoidance pain	“I want to do what normal teenagers do and not be reminded of my illness all the time. It keeps my mind off the pain and makes me forget about medication and physiotherapy every now and then. Also people think I am perfectly healthy when I participate in normal things—and that helps me forget about it” (female, JIA) [43]

## Symptom experience

### Fluctuating pain

Adolescents with JIA and CIPS identified pain as the defining characteristic of their disease [41, 54, 61]. The intensity of pain was described as both constant and varying, such that, although pain was constantly present and experienced at a daily background level, there were periods of heightened perception of pain intensity which could occur regularly or with no logical cause [59, 60].

In one study of CIPS, participants referred to these high levels of pain as ‘pain attacks’ or ‘flare-ups’, which could occur several times a day and last for hours [60]. Similarly, the term ‘flare-up’ was used in seven JIA studies, though this time referring to periods of acute disease [37, 38, 46, 49, 53, 54, 56]. Little differences were observed between the experiences of disease and pain flares, with both reported to negatively impact adolescents’ lives, including their level of confidence, identity and independence in daily living and social activities [37]. They were unpredictable, with participants not knowing what activity might cause intensified disease or pain [37, 60]. Participants described rest, lying down, avoiding activities and using aids such as crutches to manage flares, but when suspected to be a disease flare in JIA, the hospital team were contacted for advice and for potential change in medication [49, 53, 56, 60].

### Causes of fluctuating pain

Adolescents reported feelings of uncertainty about why pain increases [33, 59, 61, 62], but expressed interest in knowing what precipitated them and how to prevent them [54]. In JIA, increased pain was sometimes indicative of disease activity [31, 32, 41], particularly among older adolescents who believed pain to be caused by their internal pathology (e.g. inflammation) [32]. Younger children showed greater difficulties understanding and conceptualising their pain [31, 32] which may be due to cognitive immaturity and not having learnt how to label their experience [37]. In other cases, the cause of increased pain was not perceived by them to be the arthritis, but a result of their behaviour (e.g. overdoing activities), emotions (e.g. stress) and external factors (e.g. cold weather) [33, 49, 61]. For others, particularly young people with CIPS, they were unable to relate the pain to any specific event [60], with pain suddenly occurring “for no reason” [61]. This unpredictability and uncertainty of not knowing what might cause intensified pain appeared to be a source of concern for adolescents with both arthritis and CIPS [59–61].

### Fatigue

Pain and fatigue were found to enter into a feedback loop in which an increase in pain at night disturbed the quantity and quality of adolescents’ sleep and led to fatigue in the morning [48, 52]. Daytime fatigue, in turn, was found to exacerbate pain which further disrupts sleep at night [52].

### Psychological distress

Adolescents’ lives were controlled by the uncertainty related to pain [48]. In periods of stability, adolescents saw themselves as normal [34], but getting new pain, or having changes in pain, could lead to feelings of sadness and anxiety especially when it struck with no logical cause [39, 51]. As a result, young people described emotionally oscillating between feelings of hope to feelings of despair that they had lost control of their pain [39]. It was difficult for young people to be spontaneous which compromised their ability to engage in daily activities and set them apart from their peers. This had a considerable emotional and psychological impact on adolescents as it led to feelings of isolation, difference, and decreased competence [47].

## Disruption and loss

### Restricted body

All adolescents have to learn to live with a restrictive body which can be described as ‘feeling like a ticking fire bomb’, where suddenly ‘the worst case’ could happen [48]. They have to put adjustments in place throughout the day to accommodate and reduce the event of an increase in pain [36], however, this increased feelings of being different from peers and set up barriers to what they want to do [36].

### Loss of social life

During times of pain worsening, adolescents describe difficulties engaging in normal social experiences such as sports and going to friends’ houses [46, 47, 50, 58, 59, 61], and report impacts on school attendance, concentration and their engagement in school activities [39, 48, 49, 59–61]. Being able to participate in education and leisure activities were seen as features of normal adolescence, and an inability to do so may lead to feelings of being different, social isolation, and difficulty building and maintaining an extensive social network [35, 43, 48, 63, 64].

### Future uncertainty

Adolescents expressed concerns about how long the pain would last, when it would recur, how it would affect their plans and the long-term prospects of requiring medication [39, 47, 53, 55, 61, 62]. Concerns about the future appeared to be more significant for older compared with younger adolescents [36, 39, 47, 53, 55] who may be more aware of the implications on their lives due to greater abstract thought in middle to late adolescence [32]. Concerns were also expressed more in those who had experienced pain for a shorter period, suggesting that those with longer pain duration may normalise their experiences and learn to adapt their lives to their pain [47].

### Disbelieved and stigmatised

Adolescents faced issues with legitimacy with peers [47, 53, 57], teachers [39, 53], siblings [50] and professionals [47, 60, 62] when there were no visible signs of pain or disease. Particularly among adolescents with CIPS, when professionals were unable to find an organic cause to their condition and referred them to a psychologist, they felt that the pain was perceived to be imagined [60]. This was further compounded by the unpredictable and fluctuating nature of pain, which makes it difficult for others to understand the nature of pain [44, 48, 53]. Individuals took steps to control feelings of stigmatisation, such as choosing not to disclose their pain as they perceived that nobody would understand or believe them [43, 56].

## Regaining control

### Medication and therapies

The fluctuating nature of pain was problematic because it could affect adolescents’ need for medication and other therapies [51]. Pharmacological treatments were often the first treatment option for pain but could be painful if given by injection, last only a short duration, have unpleasant side effects, and interrupt daily routines [36, 48, 61]. Among the non-pharmacological measures, physical activity was mentioned the most, with some deeming it to be effective at relieving pain and stiffness [39, 44, 57, 58], while others believing it to be an aggravating factor of pain [31, 49]. Those that viewed physical activity as an aggravating factor of pain were more likely to be newly diagnosed with a pain disorder [31, 46], who may not understand that physical activity does not provide immediate pain relief but it is more effective in the long term, as recognised by adolescents who have greater experience of the disease.

### Social support

Positive social support appeared to have a buffering effect on coping with pain [48]. Adolescents relied heavily on their parents for practical and emotional support when they experienced severe pain [40, 60], and turned to friends for helping them maintain a sense of normality [34, 48]. Schools were important for providing practical assistance by means of excusing adolescents from physical education, allowing them to photocopy notes, stay inside during cold weather, and providing extra time to get to their next class [36, 39, 53, 60].

### Adapting behaviours

Older adolescents and those who had experienced pain for longer come to accept that pain is a part of their identity and adapt their lives to the condition [45–47]. To deal with challenges related to unpredictability, they report employing behaviours to resist or accommodate pain including rest, pacing, planning activities and using carefully structured daily routines [31, 43, 47, 48, 56, 58, 61].

### Cognitive strategies

Acquiring knowledge and understanding symptom manifestations were critical aspects in learning to effectively cope with the pain, and helped overcome feelings of helplessness and distress when increased pain was experienced [44, 48, 53]. Engaging in positive thinking also helped minimise the significance of pain [34, 36, 45, 55, 59], as well as mentally preparing themselves for pain when they ‘overdo’ activities [48, 61], and ignoring the pain since ‘there is nothing you can do about it’ [34].

### Avoiding pain

Concealing or minimising pain was common, where adolescents refrained from telling their parents about pain to prevent them from becoming anxious or from hassling them [47, 60], and from their peers in order to maintain a positive social identity [36, 46, 48, 55, 59, 62]. Those who were more likely to conceal their pain were in the early to middle phases of adolescence [46, 56], while older adolescents were more open about their condition and avoided activities they perceive as potentially pain triggering [36].

## Discussion

Adolescents with chronic musculoskeletal pain experience a daily background of symptoms which frequently fluctuate. When pain is stable, adolescents perceive themselves to be normal and lead lives like their healthy peers. But during unstable periods, that is, periods of heightened perception of symptoms, pain imposed restrictions on their school and social activities which led to a sense of isolation and difference from their peers. Adolescents experienced stigma and misunderstanding from others who have little understanding of how pain can rapidly change, and expressed fear and uncertainty about pain and how it will affect the future. Adolescents implemented various strategies to overcome challenges imposed by unpredictable fluctuations and regain a sense of control.

Our findings are comparable with a recent systematic review which reports six themes in which JIA impacted young people’s lives: aversion to being different, striving for normality, stigma and misunderstanding, suspension in uncertainty, managing treatment and a desire for knowledge [65]. In contrast to the latter review, we also found that experiences of pain can be influenced by adolescents’ developmental status, pain condition, and duration of pain experience:

### Developmental status

Differences were apparent based on adolescents’ developmental status. Feelings of frustration were expressed more strongly by older adolescents, who had greater understanding of the internal physical pathology of pain and were aware of the implications on their lives [32, 36, 39, 53]. To them, pain was a reminder of their disabling condition and the potential ramifications on their future, including their school performance, vocational opportunities, and relationship with peers. This greater understanding of pain and its limitations have been found to be a reason why older adolescents with JIA report higher levels of pain than their younger peers [32].

Developmental stage also impacted on the way adolescent’s responded to pain, with early to middle age adolescents more likely to internalise or suppress their pain in order to maintain a positive social identity [56]. This is consistent with brain development during this time, in which the prefrontal cortex, which is the site of mature functioning such as long-term planning, decision making and impulse control [66, 67], is immature, with development ongoing into early twenties. Its relatively immature control during adolescence may be over-ridden by areas of the limbic system which mature faster and are involved in reward seeking, risk taking and peer interaction [66, 67]. Thus at times of excitement with peers or during crises such as flare, adolescents may regress from abstract to concrete thinking [68] and are less likely to think rationally and cope effectively with their pain [67–70]. This can have consequences for later life as untreated pain in childhood and adolescence can lead to chronic pain in adulthood [71]. A developmentally appropriate approach to pain communication and management is required which considers the impact of biological, psychological, social and vocational factors on individuals’ level of understanding. This includes using ‘here and now’ concrete explanations and avoiding more abstract ‘if and when’ communications [72]. The skills needed to communicate with young people will vary across all age spectrums, and paediatric, adolescents and adult rheumatologists can benefit from deeper understanding of these differences.

### Musculoskeletal pain condition

Adolescents with JIA and CIPS described difficulties in their search for a diagnosis and gaining empathy and understanding for invisible pain. These broader level findings of uncertainty, not being believed by others and stigma are comparable with young people’s experiences of other long-term conditions (e.g. epilepsy, renal failure, inflammatory bowel disease [73–75]), suggesting that attempts to address challenges and disruptions from chronic illnesses can be considered within the same framework [76]. However these difficulties appeared to be more pronounced in young people with CIPS who also faced greater issues with the perception that pain was entirely psychological or ‘made-up’. Assessing and managing CIPS can be more challenging and demanding across all age spectrums and part of diagnosing involves ruling out other inflammatory joint conditions which can be frustrating for young people when faced with ‘physical symptoms’ without a specific treatable medical diagnosis. As a result, participants appear to have greater mistrust in medical providers who could not see or diagnose the cause and had lowered expectations of physician involvement. The perception that pain is undervalued by health professionals has been reported by Lee et al. [77] who found that healthcare professionals had a biomedical understanding of pain, with the assumption that if inflammation and disease processes are managed, the symptom of pain will improve. Improved training for healthcare professionals, including rheumatologists, nurses, physical therapists and occupational therapists, to move beyond a medical model of pain is needed to assist in affirming adolescents’ experiences and improve the quality and outcome of clinical relationships. This will include understanding biopsychosocial influences and gaining knowledge in co-morbid conditions that can worsen pain (e.g. anxiety, depression).

### Pain duration

Adolescents who are older at pain onset, or have experienced pain for a shorter duration, perceive pain to be greater and find it difficult to adjust to the functional changes and develop effective coping strategies [55, 78]. But as they mature and experience pain for longer, they develop an increasing degree of comfort with their own individuality, normalise their experiences and adapt their lives to their abilities [37, 45–47]. They may also develop tolerance to living with pain the longer they experience it [79] which allows them to push through the pain to achieve their goals e.g. playing sport. In this context, being able to participate in school and social activities were seen as vital to being normal and added to their social identity, which suggests a psychological influence may be present which modulates the perception of pain.

## Future directions

It is a matter of concern that adolescents expressed difficulty and disruption related to having pain despite advances in medical management. It was reported that adolescents faced increased challenges during unstable periods, however, there was a lack of differentiation between whether these periods occurred as part of their ‘typical’ pain fluctuations, or whether they were symptom exacerbations colloquially referred to as ‘flares’. Pain flares have been found, in adult musculoskeletal conditions, to be a complex, multi-layered, whole-body experience that affect quality of life [16–18, 80, 81]. There is currently no published literature on pain flares in the paediatric literature, and clinical measures used to identify disease flares (e.g. inflammatory markers, active joints, etc.) have limited value due to the lack of consistent correlation between pain and inflammation in JIA and CIPS [5, 7]. Further research is needed to explore how pain flares are defined and experienced in young people, and how they differ from their normal fluctuations in pain. Individual factors such as developmental status, pain condition, and duration of pain experience would be important determinants to take into account as they can impact how pain is perceived and experienced.

## Limitations

First, the review covered 35 years of research where pain and disease management has changed significantly. Table 2 shows the studies presented in chronological order, but little difference is observed in experiences of pain despite advances in treatment. Second, we included studies with broad age-ranges, which included participants that fall out of the age-range for adolescence. While there were efforts to account for differences based on age and developmental status across some studies [31–33, 36, 39, 46, 47, 49, 52, 53, 55–58], others overlooked the potential influence of these factors on their findings. Third, the review included five studies with pain syndromes other than JIA and CIPS (e.g. headache) [47, 50, 52, 57, 59] and two studies which included chronic conditions not specially related to pain (e.g. asthma) [34, 40]. Of these, we were unable to determine which themes were derived from our target sample in four studies [40, 47, 50, 52], however, two of these reported no evidence of differences across diagnostic groups [47, 52] suggesting that adolescents share many of the same experiences and concerns regardless of the type of illness. Fourth, the studies were conducted in 11 different countries, therefore it is likely that experiences of pain can be influenced by geographical location, culture, race, and ethnicity (e.g. different countries have different pressures to health systems which can affect waiting times for assessment, diagnosis and management) [42, 54, 56]. These views were not analysed separately in this paper.

## Conclusions

The review revealed that adolescents with chronic musculoskeletal pain live with a daily background level of symptoms which frequently fluctuate. It was not clear whether these symptoms and challenges were experienced as part of ‘typical’ fluctuations in pain, or whether they reflect symptom exacerbations classified as ‘flares’. Further research is needed to explore the frequency and characteristics of pain flares, and how they differ from their typical fluctuations in pain. The review also highlighted that pain perception and experience were influenced by adolescents’ developmental status, pain condition, and duration of pain experience. It is important that parents and health professionals understand the impact of these differences on young people’s experiences in order to facilitate plans for a more personalised approach to pain management.

## Supplementary information

**Additional file 1.**

## Data Availability

Not applicable.

## Abbreviations

CIPS: Chronic Idiopathic Pain Syndromes
CRPS: Complex Regional Pain Syndrome
JIA: Juvenile idiopathic arthritis
PEO: Population, Exposure, Outcome
