# Supplementary material for: Adolescents’ experiences of fluctuating pain in musculoskeletal disorders: a qualitative systematic review and thematic synthesis
Source: BMC Musculoskelet Disord. 2020 Oct 2;21:645. doi: 10.1186/s12891-020-03627-1 (PMC7532580; doi:10.1186/s12891-020-03627-1)
Supplement: Supplementary file 1 — Additional file 1. [file 12891_2020_3627_MOESM1_ESM.docx]

Additional file 1.

Search strategy and results for each database (Carried out on 04/03/18)

| **#** | **Search History** | **Results** |
| --- | --- | --- |
| **Medline (OVID). 1946 to February week 5 2018** |  |  |
| 1 | adolescent | 1854415 |
| 2 | child or children | 2019020 |
| 3 | young adult* | 685920 |
| 4 | young people | 19294 |
| 5 | youth | 48835 |
| 6 | teenager | 1959 |
| 7 | 1 or 2 or 3 or 4 or 5 or 6 | 3293392 |
| 8 | juvenile rheumatoid arthritis | 2988 |
| 9 | juvenile chronic arthritis | 1025 |
| 10 | juvenile idiopathic arthritis | 3607 |
| 11 | juvenile arthritis | 742 |
| 12 | Juvenile fibromyalgia | 58 |
| 13 | fibromyalgia | 9010 |
| 14 | chronic idiopathic pain | 19 |
| 15 | generalised pain | 29 |
| 16 | widespread pain | 1131 |
| 17 | diffuse* pain | 245 |
| 18 | musculoskeletal pain | 5179 |
| 19 | idiopathic pain | 100 |
| 20 | 8 or 9 or 10 or 11 or 12 or 13 or 14 or 15 or 16 or 17 or 18 or 19 | 22125 |
| 21 | pain | 542007 |
| 22 | flare or pain flare | 8155 |
| 23 | fluctuat* | 73253 |
| 24 | exacerbat* | 76590 |
| 25 | Variability or variable | 457502 |
| 26 | 21 or 22 or 23 or 24 or 25 | 1130766 |
| 27 | qualitative | 160041 |
| 28 | interview | 126783 |
| 29 | focus group | 15165 |
| 30 | 27 or 28 or 29 | 282148 |
| 31 | 7 and 20 and 26 and 30 | 178 |
| **Embase (OVID). 1974 to 2018 March 04** |  |  |
| 1 | adolescent/ | 1458584 |
| 2 | child or children | 2260573 |
| 3 | young adult* | 295091 |
| 4 | young people | 29894 |
| 5 | youth | 63337 |
| 6 | 1 or 2 or 3 or 4 or 5 | 3137346 |
| 7 | juvenile rheumatoid arthritis | 18063 |
| 8 | juvenile chronic arthritis | 1315 |
| 9 | juvenile idiopathic arthritis | 8594 |
| 10 | juvenile arthritis | 1422 |
| 11 | Juvenile fibromyalgia | 125 |
| 12 | fibromyalgia | 18630 |
| 13 | chronic idiopathic pain | 27 |
| 14 | generalised pain | 52 |
| 15 | widespread pain | 2218 |
| 16 | diffuse* pain | 471 |
| 17 | musculoskeletal pain | 11418 |
| 18 | idiopathic pain | 167 |
| 19 | 7 or 8 or 9 or 10 or 11 or 12 or 13 or 14 or 15 or 16 or 17 or 18 | 48912 |
| 20 | pain | 1045815 |
| 21 | flare or pain flare | 15980 |
| 22 | fluctuat* | 100433 |
| 23 | exacerbat* | 165713 |
| 24 | Variability or variable | 891748 |
| 25 | 20 or 21 or 22 or 23 or 24 | 2151272 |
| 26 | qualitative | 245067 |
| 27 | interview | 280570 |
| 28 | focus group | 22823 |
| 29 | 26 or 27 or 28 | 484889 |
| 30 | 6 and 19 and 25 and 29 | 246 |
| 31 | Limit 30 to human | 236 |
| 32 | Limit 31 to English | 227 |
| **PsycINFO (OVID). 1806 to February 2018** |  |  |
| 1 | adolescent | 148364 |
| 2 | child or children | 617813 |
| 3 | young adult* | 43780 |
| 4 | young people | 24900 |
| 5 | youth | 83886 |
| 6 | teenager | 1343 |
| 7 | 1 or 2 or 3 or 4 or 5 or 6 | 778196 |
| 8 | juvenile rheumatoid arthritis | 186 |
| 9 | juvenile chronic arthritis | 19 |
| 10 | juvenile idiopathic arthritis | 114 |
| 11 | juvenile arthritis | 64 |
| 12 | Juvenile fibromyalgia | 22 |
| 13 | fibromyalgia | 3033 |
| 14 | chronic idiopathic pain | 19 |
| 15 | generalised pain | 4 |
| 16 | widespread pain | 411 |
| 17 | diffuse* pain | 43 |
| 18 | musculoskeletal pain | 1407 |
| 19 | idiopathic pain | 75 |
| 20 | 8 or 9 or 10 or 11 or 12 or 13 or 14 or 15 or 16 or 17 or 18 or 19 | 4815 |
| 21 | pain | 89522 |
| 22 | flare or pain flare | 351 |
| 23 | fluctuat* | 16439 |
| 24 | exacerbate or exacerbation | 8212 |
| 25 | Variability or variable | 128197 |
| 26 | 21 or 22 or 23 or 24 or 25 | 237085 |
| 27 | qualitative | 140567 |
| 28 | interview | 172941 |
| 29 | focus group | 15588 |
| 30 | 27 or 28 or 29 | 300583 |
| 31 | 7 and 20 and 26 and 30 | 58 |
| **CINAHL Plus (EBSCOhost). 1937 to inception** |  |  |
| 1 | adolescent | 101289 |
| 2 | child or children | 550329 |
| 3 | young adult* | 187,437 |
| 4 | young people | 14079 |
| 5 | youth | 33771 |
| 6 | teenager | 4842 |
| 7 | 1 or 2 or 3 or 4 or 5 or 6 | 757531 |
| 8 | juvenile rheumatoid arthritis | 2479 |
| 9 | juvenile chronic arthritis | 121 |
| 10 | juvenile idiopathic arthritis | 1609 |
| 11 | juvenile arthritis | 2837 |
| 12 | Juvenile fibromyalgia | 67 |
| 13 | fibromyalgia | 5669 |
| 14 | chronic idiopathic pain | 74 |
| 15 | generalised pain | 44 |
| 16 | widespread pain | 958 |
| 17 | diffuse* pain | 375 |
| 18 | musculoskeletal pain | 3553 |
| 19 | idiopathic pain | 342 |
| 20 | 8 or 9 or 10 or 11 or 12 or 13 or 14 or 15 or 16 or 17 or 18 or 19 | 12820 |
| 21 | pain | 230802 |
| 22 | flare or pain flare | 1964 |
| 23 | fluctuat* | 6369 |
| 24 | exacerbat* | 17088 |
| 25 | Variability or variable | 171741 |
| 26 | 21 or 22 or 23 or 24 or 25 | 412304 |
| 27 | qualitative | 121833 |
| 28 | interview | 236479 |
| 29 | focus group | 40997 |
| 30 | 27 or 28 or 29 | 298233 |
| 31 | 7 and 20 and 26 and 30 | 117 |
